# NOVEL GENOMIC AND EXCEPTIONAL LONGEVITY FINDINGS FROM THE LONG LIFE FAMILY STUDY

**DOI:** 10.1093/geroni/igac059.1554

**Published:** 2022-12-20

**Authors:** Mary Wojczynski, Nancy W Glynn, Evan Hadley

## Abstract

The Long Life Family Study (LLFS), funded by the National Institute on Aging, is an international collaborative study of the genetics and familial components of exceptional longevity and healthy aging. We phenotyped 4,953 individuals from 539 two-generational families (1,727 proband; 3,226 offspring) at baseline (2006-2009). A second visit (2014-2017) was conducted for 2,904 (478 proband; 2,426 offspring) participants. The longitudinal, comprehensive in-person visits measured domains of healthy aging, including physical performance, cognition, and blood markers. Extensive genetic analyses were performed using the baseline blood draw, including genotyping with the Illumina 2.5M Human Omni array, linkage analyses with the families, whole genome sequencing using the TopMED protocol, and metabolomic assays. Collectively, this symposium will present novel findings that examined differences in end of life events and cause of death between non-exceptional and exceptional long-lived women, elucidate potential rare variants associated with exceptional longevity, and examine new potential metabolomics pathways involved in gait speed and cardiovascular disease. Specifically, Dr. Galvin will share results from three Danish nationwide studies (including LLFS) on different end of life events in long-lived female siblings. Then, Dr. Gurinovich will share findings on new uncommon variants associated with extreme longevity. Next, Dr. Kuipers will discuss associations between lipid metabolomics and vascular health. Lastly, Dr. Santanasto will discuss lipid metabolomics associated with lower odds of slow gait speed. Dr. Evan Hadley, NIA, will be the discussant and will share insights and propose future directions for LLFS.

